# Chondrodysplasias With Multiple Dislocations Caused by Defects in Glycosaminoglycan Synthesis

**DOI:** 10.3389/fgene.2021.642097

**Published:** 2021-06-16

**Authors:** Johanne Dubail, Valérie Cormier-Daire

**Affiliations:** ^1^Université de Paris, INSERM UMR 1163, Institut Imagine, Paris, France; ^2^Service de Génétique Clinique, Centre de Référence Pour Les Maladies Osseuses Constitutionnelles, AP-HP, Hôpital Necker-Enfants Malades, Paris, France

**Keywords:** chondrodysplasia, dislocations, glycosaminoglycan, congenital disorder of glycosylation, genotype-phenotype correlation

## Abstract

Chondrodysplasias with multiple dislocations form a group of severe disorders characterized by joint laxity and multiple dislocations, severe short stature of pre- and post-natal onset, hand anomalies, and/or vertebral anomalies. The majority of chondrodysplasias with multiple dislocations have been associated with mutations in genes encoding glycosyltransferases, sulfotransferases, and transporters implicated in the synthesis or sulfation of glycosaminoglycans, long and unbranched polysaccharides composed of repeated disaccharide bond to protein core of proteoglycan. Glycosaminoglycan biosynthesis is a tightly regulated process that occurs mainly in the Golgi and that requires the coordinated action of numerous enzymes and transporters as well as an adequate Golgi environment. Any disturbances of this chain of reactions will lead to the incapacity of a cell to construct correct glycanic chains. This review focuses on genetic and glycobiological studies of chondrodysplasias with multiple dislocations associated with glycosaminoglycan biosynthesis defects and related animal models. Strong comprehension of the molecular mechanisms leading to those disorders, mostly through extensive phenotypic analyses of *in vitro* and/or *in vivo* models, is essential for the development of novel biomarkers for clinical screenings and innovative therapeutics for these diseases.

## Introduction

In 2019, the Nosology Committee of the International Skeletal Dysplasia Society published a new edition of the Nosology and Classification of Genetic Skeletal Disorders (Mortier et al., 2019). This 2019 version covers 461 different diseases divided into 42 groups according to their clinical, radiographic, and/or molecular phenotypes.

The application of massively parallel sequencing technology has led to the discovery in the last few years of a great number of genetic defects responsible for skeletal disorders. To date, the molecular bases have been identified for 425/461 (92%) of these disorders. In total, pathogenic variants affecting 437 different genes encoding enzymes, extracellular matrix (ECM) proteins, membrane transporters, cilia proteins, signal transduction proteins, and transcription factors have been found.

**Figure 1:**
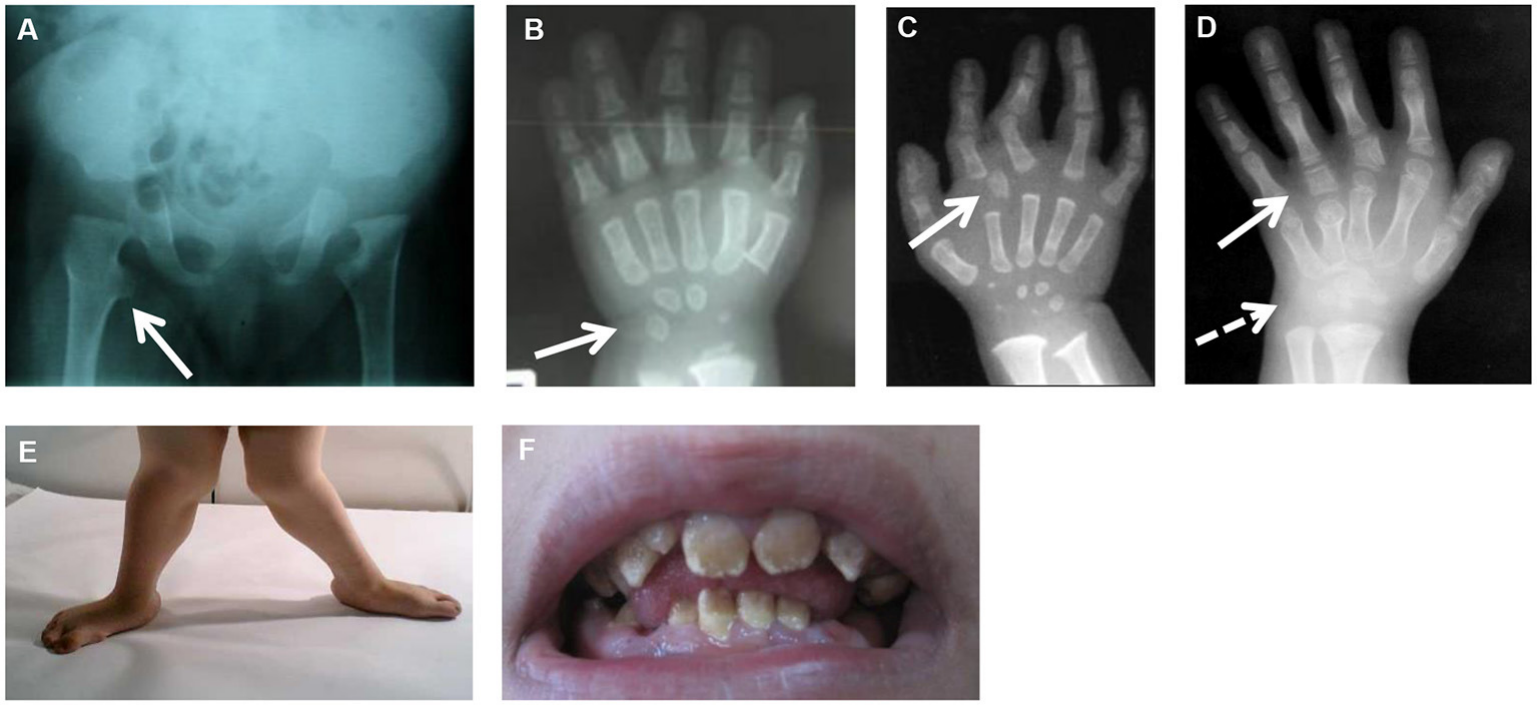
Common and specific clinical features in CMD. **(A)** Hip X-rays showing a monkey wrench appearance of femur (see arrow). **(B)** Hand X-rays of a patient with *XYLT1* mutations at 8 months of age showing advanced carpal ossification (see arrow). **(C)** Hand X-rays of a patient with *CANT1* mutations at 1 year of age showing presence of a delta phalanx (see arrow). **(D)** Hand X-rays of a patient with *IMPAD1* mutations at 5 years of age showing presence of hyperphalangy (see plain arrow) and carpal synostosis (see dashed arrow). **(E)** Genu valgum due to joint laxity. **(F)** Amelogenesis imperfecta in a patient with *SLC10A7* mutations.

In this review, we will be focusing on the skeletal dysplasias caused by defects in the glycosaminoglycan (GAG) biosynthesis and, more specifically, on the group of chondrodysplasias with multiple dislocations (CMD), listed in groups 20, 4, and 25 in the International Classification on Genetic Skeletal Disorders (Mortier et al., 2019). They form a group of severe disorders characterized by joint laxity and multiple dislocations affecting large joints (such as hip, knee, and shoulder), severe short stature of pre- and post-natal onset, hand anomalies, and/or vertebral anomalies. Common radiographic features include advanced carpal and tarsal bone age and exaggerated trochanters giving a monkey wrench appearance of the femoral neck (Figure 1). Additional skeletal features, for instance, epiphyseal or metaphyseal changes, specific facial dysmorphisms, and cleft palate, are often part of the clinical presentation. A variable combination of other clinical features such as loose or old-appearing skin, congenital heart defects, teeth anomalies, intellectual disabilities, and obesity can also be observed in those patients. Up to now, more than 25 syndromes with autosomal recessive inheritance patterns have been described (Table 1). CMD has mostly been linked to pathogenic variants in genes implicated in the biosynthesis of proteoglycan (PG). PGs are large macromolecules that are widely expressed in multicellular organisms, present in the ECM or at the cell surface. They consist of a core protein and one or more covalently linked polysaccharides, called GAGs. GAGs are large linear polysaccharides composed of repeated disaccharide units consisting of amino sugar, either *N*-acetylglucosamine (GlcNAc) or *N*-acetylgalactosamine (GalNAc), and uronic acid, either glucuronic acid (GlcUA) or iduronic acid (IdoUA), except keratan sulfate (KS) in which disaccharide units consist of GlcNAc and galactose. Hyaluronan (HA) is a non-sulfated glycosaminoglycan and is not attached to any core protein. It is synthesized by a specific synthesis pathway taking place at the cell membrane. Sulfated GAGs are classified into four groups based on the composition of their disaccharide units: chondroitin sulfate (CS), dermatan sulfate (DS), KS, and heparan sulfate. In addition, these GAGs undergo further modifications, such as sulfation at various positions of the chain and epimerization of uronic acid (Lindahl et al., 2017).

**Table 1:** Skeletal dysplasias caused by defects in GAG biosynthesis and related animal models.

	**Human**				**Mouse**		**Zebrafish/xenopus**	
	**Clinical entities *(MIM/inheritance)***	**Main skeletal features**	**Others clinical features**	**Specific features**	**Genotype**	**Main phenotype**	**Genotype**	**Main phenotype**
**Linker synthesis**								
*XYLT1*	Desbuquois dysplasia type 2 *(MIM: 615777/AR)* Bui et al., 2014; Schreml et al., 2014; Jamsheer et al., 2016; Silveira et al., 2016; Al-Jezawi et al., 2017; Guo et al., 2017a; LaCroix et al., 2019	Dislocation of large joints with generalized joint laxity, severe pre- and post-natal growth retardation, flat face, short, long bones, and advanced carpal and tarsal ossification	Cleft palate, developmental delay, truncal obesity		*Pug* mice Mis et al., 2014	Early chondrocyte maturation and early ossification leading to disproportionate dwarfism	*xylt^−^* mutant zebrafish Eames et al., 2011	Altered craniofacial skeletal morphology, decreased cartilage matrix, and increased perichondral bone
*XYLT2*	Spondylocular syndrome *(MIM: 605822/AR)* Munns et al., 2015; Taylan et al., 2016, 2017; Umair et al., 2018; Guleray et al., 2019; Kausar et al., 2019	Facial dysmorphism, short trunk, platyspondyly and osteoporosis	Ocular defects, cardiac septal defect	Osteoporosis, cataracts, renal detachment, hearing loss	*Xylt2^−/−^*mice Condac et al., 2007; Sivasami et al., 2019; Ferencz et al., 2020	Post-natal liver and kidney cysts, adipose tissue loss, increased heart, spleen, and lung weight	N.D	N.D.
*FAM20B*	Neonatal short limb dysplasia *(MIM: –/AR)* Kuroda et al., 2019	Very short stature, multiple dislocations of large joints, midface hypoplasia, and thoracic hypoplasia	Respiratory failure	Mesomelic shortening, preaxial digital hypoplasia	*Fam20b^−/−^*mice Vogel et al., 2012	Lethal during embryonic period with multiorgan hypoplasia	*Fam20b^−^* mutant zebrafish Eames et al., 2011	Altered craniofacial skeletal morphology, decreased cartilage matrix, and increased perichondral bone
					*Osr2-Cre;Fam20b^*fl*/*fl*^* mice Ma et al., 2016	Chondrosarcoma and post-natal ossification defects		
					*Wnt1-Cre;Fam20b ^*f*/*fl*^* mice Liu et al., 2018	Multiple craniofacial defects, including complete cleft palate leading to post-natal death		
					*K14-Cre;Fam20b ^*f*/*fl*^* mice Tian et al., 2015	Supernumerary incisors		
					*Col1a1-Cre;Fam20b ^*f*/*fl*^* mice Saiyin et al., 2019	Growth retardation and spine deformity		
*B4GALT7*	Ehlers-Danlos syndrome (EDS) progeroid variant or EDS spondylodysplastic type 1 (EDSSPD1), including Larsen syndrome, la Reunion variant *(MIM: 130070/AR)* Okajima et al., 1999; Faiyaz-Ul-Haque et al., 2004; Guo et al., 2013; Cartault et al., 2015; Salter et al., 2016; Ritelli et al., 2017; Mihalic Mosher et al., 2019	Short stature, hypermobile joints, generalized osteopenia, craniofacial dysmorphism	Loose but elastic skin, defective wound healing, hypotonic muscle		N.D.	N.D.	*b4galt7* morphant zebrafish, *b4galt7^*Cas*9/*sgRNA*^* crispant zebrafish Delbaere et al., 2020	Short stature, deformed pectoral fins, craniofacial dysmorphism, reduced mineralization
*B3GALT6*	Spondyloepimetaphyseal dysplasia with joint laxity, Beighton type *(MIM: 271640/AR)* or EDS spondylodysplastic type 2 (EDSSPD2) (*MIM: 615349/AR*) Malfait et al., 2013; Nakajima et al., 2013; Ritelli et al., 2015; Vorster et al., 2015; Alazami et al., 2016; Trejo et al., 2017; Van Damme et al., 2018	Short stature, joint laxity, epimetaphyseal dysplasia, severe kyphoscoliosis, craniofacial dysmorphism, and osteopenia	Loose skin, defective wound healing, hypotonic muscles		N.D.	N.D.	N.D.	N.D.
*B3GAT3*	Larsen-like syndrome *(MIM: 245600/AR)* Baasanjav et al., 2011; von Oettingen et al., 2014; Budde et al., 2015; Jones et al., 2015; Alazami et al., 2016; Job et al., 2016; Bloor et al., 2017; Yauy et al., 2018; Colman et al., 2019; Ritelli et al., 2019	Multiple dislocations of large joints, short stature, craniofacial dysmorphism	Congenital heart defects		*B3gat3^−/−^* mice Izumikawa et al., 2010	Very early embryonic lethality due to cytokinesis failure	*b3gat3^−/−^* mutant zebrafish Holmborn et al., 2012	CS synthesis abolished, abnormal pharyngeal cartilage morphogenesis
**CS/DS chain elongation**								
*CSGALNACT1*	Joint dislocations and skeletal dysplasia, Desbuquois-like *(MIM: 618870/AR)* Baasanjav et al., 2011; Vodopiutz et al., 2017; Mizumoto et al., 2020	Nonproportionate short stature, hyperlordosis, advanced bone age, mild joint laxity			*Csgalnact1^−/−^* mice Watanabe et al., 2010; Sato et al., 2011; Yoshioka et al., 2017	Slight dwarfism Abnormal perineural net and behavior	N.D.	N.D.
*CSGALNACT2*	N.D.	N.D.	N.D.		*Csgalnact2^−/−^* mice Shimbo et al., 2017	Normal development, fertility, and growth rates	N.D.	N.D.
					*Csgalnact1^−/−^*; *Csgalnact2^−/−^* mice Shimbo et al., 2017	Severe dwarfism and post-natal lethality		
*CHSY1*	Temtamy preaxial brachydactyly syndrome (TPBS) *(MIM: 605282/AR)* Li et al., 2010; Tian et al., 2010; Sher and Naeem, 2014	Growth retardation, bilateral and symmetric preaxial brachydactyly and hyperphalangism of digits, joint laxity, facial dysmorphism, dental anomalies	Delayed motor and mental development, sensorineural hearing loss	Hyperphalangism and preaxial brachydactyly	*Chsy1^−/−^* mice Wilson et al., 2012	Chondrodysplasia, decreased bone density, and profound digit patterning defects	*chsy* morphant zebrafish Li et al., 2010	Reduced body length, compromised pectoral fin formation, cranial dysmorphism, inner ear formation defects
*CHPF*	N.D.	N.D.	N.D.		*Chpf^−/−^* mice Ogawa et al., 2012	No overt morphological phenotype	N.D.	N.D.
*CHPF2*	N.D.	N.D.	N.D.		N.D.	N.D.	N.D.	N.D.
*DSE*	Ehlers-Danlos syndrome musculocontractural type 2 *(MIM: 615539/AR)* Müller et al., 2013; Syx et al., 2015; Lautrup et al., 2020	Joint dislocation and deformities, distinct craniofacial features	Skin hyperextensibility, bruisability and fragility, multiple congenital contractures		*Dse^−/−^* mice Maccarana et al., 2009; Gustafsson et al., 2014	Smaller, with a 30% reduced body weight and kinked tail at birth, altered skin morphology and skin tensile strength, abdominal wall defect	*dse* morphant xenopus Gouignard et al., 2016	Abnormal development of neural crest-derived structures
*DSEL*	N.D.	N.D.	N.D.		*Dsel^−/−^* mice Bartolini et al., 2012	No overt morphological phenotype		
					*Dse^−/−^; Dsel^−/−^* mice Stachtea et al., 2015	Perinatal lethality with developmental defects		
**HS chain elongation**								
*EXTL1*	N.D.	N.D.	N.D.		N.D.	N.D.	N.D.	N.D.
*EXTL2*	N.D.	N.D.	N.D.		*Extl2^−/−^* mice Nadanaka et al., 2013a; Purnomo et al., 2013; Pu et al., 2020	Increased GAG synthesis affecting liver regeneration, aorta calcification and axonal loss in induced disease models	N.D.	N.D.
*EXTL3*	Immunoskeletal dysplasia with neurodevelopmental abnormalities (ISDNA) *(MIM: 617425/AR)* Guo et al., 2017b; Oud et al., 2017; Volpi et al., 2017	Severe platyspondyly, brachydactyly, kyphoscoliosis, facial dysmorphisms	Severe motor developmental delay, immunodeficiency linked to T-cell lymphopenia		*Extl3^−/−^* mice Takahashi et al., 2009	Embryonic lethality around 8 days post-coitum	*Extl3^−/−^* (box) mutant zebrafish Guo et al., 2017b; Oud et al., 2017; Volpi et al., 2017	Mildly altered pharyngeal cartilage morphogenesis Abnormal pectoral fin development Defective thymopoiesis
*EXT1*	Hereditary multiple exostosis type 1 *(MIM: 133700/AD) reviewed in* Pacifici, 2018	Benign osteocartilaginous tumors, especially located in metaphysis of long bones		Multiple exostosis	*Ext1^−/−^* mice Lin et al., 2000	Embryonic lethality at day 8,5 to 14,5 due to gastrulation failure	*ext1* morphant xenopus Shieh et al., 2014	Gastrulation defects
					*Ext1^*gt*/*gt*^* mice Koziel et al., 2004	Embryonic lethal, delayed hypertrophic chondrocytes differentiation leading to skeletal defects		
					*Prx-Cre; Ext1^*fl*/*fl*^* mice Matsumoto et al., 2010	Shortened and malformed limb bones, oligodactyly, and fusion of joints		
					*Gdf5-Cre; Ext1^*fl*/*fl*^* mice Mundy et al., 2011	Abnormal joint formation		
*EXT2*	Hereditary multiple exostosis type 2 (*MIM: 133701/AD*) reviewed in Pacifici (2018)	Benign osteocartilaginous tumors, especially located in metaphysis of long bones		Multiple exostosis	*Ext2^−/−^* mice Stickens et al., 2005	Embryonic lethality at day 6	*Ext2^−/−^* (dak) mutant zebrafish Norton et al., 2005; Holmborn et al., 2012; Wiweger et al., 2012	Shorter and thicker pharyngeal cartilage elements, severe truncation of pectoral fin, severe tooth formation defects
					*Ext2^+/−^* mice Stickens et al., 2005	Exostoses in ribs		
					*Ext1^+/−^ Ext2^+/−^* mice Zak et al., 2011	Exostoses in ribs and long bones		
**Sulfation**								
*SLC26A2*	Achondrogenesis type 1B *(MIM: 600972/AR)*	Fetal or perinatal lethality, extremely short extremities and trunk, micromelia			*Slc26a2^−/−^* mice and *Col2a1-cre; Slc26a2^*fl*/*fl*^* Zheng et al., 2019	Perinatal lethality, short neck with thickened soft tissue, small chest, extremely short limbs, and protuberant abdomen		
	Atelosteogenesis type 2 *(MIM: 256050/AR)*	Perinatal lethality, very short limbs, small chest, distinctive facial features, cleft palate, flattened vertebrae, cervical kyphosis, and hitchhiker's thumb		Hitchhiker's thumb				
	Diastrophic dysplasia *(MIM: 222600/AR)*	Joint dysplasia, joint pain and contractures, cleft palate, progressive scoliosis, hitchhiker's thumb	Cystic swelling of external hear	Hitchhiker's thumb	*dtd* mice Forlino et al., 2005	Reduced skeletal growth, deformities of long bones, delay in formation of secondary ossification center, long bone osteoporosis	*slc26a2* morphant zebrafish Liu et al., 2015	Abnormal otic development
	Recessive multiple epiphyseal dysplasia *(MIM: 226900/AR)* Reviewed in Bonafé et al. (1993a,b,c), Superti-Furga and Unger (1993)	Scoliosis, clubfoot, and double-layered patella						
*PAPSS1*	N.D.	N.D.	N.D.	N.D.	N.D.	N.D.	N.D.	N.D.
*PAPSS2*	Spondyloepitmetaphyseal dysplasia, Pakistani type *(MIM: 612847/AR)* Ahmad et al., 1998; Faiyaz ul Haque et al., 1998; Tüysüz et al., 2013	Short stature, short and bowed lower limbs, mild brachydactyly, enlarged knee joints, osteoarthritis, kyphoscoliosis			Brachymorphic mice Ford-Hutchinson et al., 2005	Shortened limbs, complex craniofacial phenotype, knee cartilage degeneration	N.D.	N.D.
	Brachyolmia type 1*(MIM: 271530, 271630/AR)* Miyake et al., 2012; Iida et al., 2013; Bownass et al., 2019	Short trunk, platyspondyly with irregular endplates and narrow intervertebral discs, and precocious calcification of rib cartilage	Corneal opacities					
*SLC35B2 (PAPST1)*	N.D.	N.D.	N.D.	N.D.	N.D.	N.D.	*slc35b2* (pic) mutant zebrafish Wiweger et al., 2011	Severe cartilage and bone defects, dwarfism, and craniofacial deformities
*SLC35B3 (PAPST2)*	N.D.	N.D.	N.D.	N.D.	N.D.	N.D.	N.D.	N.D.
*CHST3*	Recessive Larsen syndrome or spondyloepiphyseal dysplasia with congenital joint *(MIM: 143095/AR)* Thiele et al., 2004; Hermanns et al., 2008; van Roij et al., 2008; Tuysuz et al., 2009; Unger et al., 2010; Tanteles et al., 2013; Waryah et al., 2016; Muys et al., 2017; Srivastava et al., 2017; Albuz et al., 2020; Duz and Topak, 2020	Short stature of prenatal onset, large joint dislocations, clubfeet, kyphosis, and intervertebral disk degeneration	Minor heart valve dysplasia		*C6st1^−/−^* mice Uchimura et al., 2002	Decrease of naive T lymphocytes in spleen	N.D.	N.D.
*CHST11*	Osteochondrodysplasia, brachydactyly, and overlapping malformed digits *(MIM: 618167/AR)* Shabbir et al., 2018	Mild short stature, hand and foot malformations, predominantly brachydactyly and overlapping digits, scoliosis, dislocated patellae, and fibulae			*C4st1^*gt*/*gt*^* mice Klüppel et al., 2005	Numerous skeletal malformations, including a small rib cage, very short limbs, a twisted vertebral column, and a dome-shaped skull	N.D.	N.D.
*CHST14*	Ehlers-Danlos syndrome musculocontractural type 1 (MIM: 601776/AR) Dündar et al., 2009; Malfait et al., 2010; Miyake et al., 2010; Shimizu et al., 2011; Voermans et al., 2012; Syx et al., 2015; Sandal et al., 2018; Uehara et al., 2018, 2020; Lautrup et al., 2020	Facial dysmorphism, clubfoot, kyphoscoliosis, joint hypermobility	Contractures of thumbs and fingers, hypotonia, hyperextensible thin skin, atrial septal defect, ocular involvement	Adducted thumb, arthrogryposis	*Chst14^−/−^* mice Akyüz et al., 2013; Hirose et al., 2020	Smaller body mass, kinked tail, reduced fertility, and more fragile skin	N.D.	N.D.
*IMPAD1*	Chondrodysplasia with joint dislocations, gPAPP type *(MIM: 614078/AR)* Vissers et al., 2011; Nizon et al., 2012a	Severe growth retardation with brachydactyly and hyperphalangism with a bilateral deviation of index fingers, cleft palate, and micrognathia		Hyperphalangism, carpal synostosis, Irregular sizes of distal metacarpal epiphysis and fingers, brachymetacarpia	*Impad1^−/−^* mice Sohaskey et al., 2008	Perinatal lethality with severe dwarfism, skeletal defects, abnormal joint formation	N.D.	N.D.
**Transporter or other**								
*SLC35D1*	Schneckendecken dysplasia (SHNKND) *(MIM: 269250/AR)* Hiraoka et al., 2007; Furuichi et al., 2009; Rautengarten et al., 2019	Neonatal lethal skeletal dysplasia with extremely short long bones, small ilia, and oval-shaped vertebral bodies.		Snail-shaped ilia	*Slc35d1^−/−^* mice Hiraoka et al., 2007	Neonatal lethality, extremely short limbs flattening of vertebral bodies, hypoplasia of craniofacial bones, and short ilia	*slc35d1* morphant xenopus De Domenico et al., 2015	Lethal form of skeletal dysplasia
*SLC35A3*	Multiple congenital malformation syndrome including vertebral malsegmentation and joint dislocations *(MIM: –/AR)* Edvardson et al., 2013; Edmondson et al., 2017; Marini et al., 2017	Anomalous vertebrae, limb deformities, knee and hip dislocation	Epilepsy		N.D.	N.D.	N.D.	N.D.
*SLC10A7*	Skeletal dysplasia, osteoporosis, multiple dislocations and amelogenesis imperfecta *(MIM: 618363/AR)* Ashikov et al., 2018; Dubail et al., 2018; Laugel-Haushalter et al., 2019	Severe pre-and post-natal growth retardation, multiple dislocation, advanced carpal ossification, microretrognathia, and amelogenesis imperfecta	Heart defects, hearing loss, obesity	Amelogenesis imperfecta	*Slc10a7^−/−^* mice Dubail et al., 2018	Skeletal dysplasia, short stature, low bone density, amelogenesis imperfecta	*slc10a7* morphant zebrafish Ashikov et al., 2018	Abnormal development of several cartilage elements, strong reduction in bone mineralization
*CANT1*	Desbuquois dysplasia type 1, including Kim variant *(MIM: 251450/AR)* Huber et al., 2009; Faden et al., 2010; Furuichi et al., 2011; Nizon et al., 2012b; Inoue et al., 2014; Singh et al., 2015; Yauy et al., 2018; Menzies et al., 2019	Severe pre- and post-natal growth retardation, joint laxity, scoliosis, and advanced carpal ossification with presence of a delta phalanx		Bifid distal phalanx of thumb/delta phalanx	*Cant1^−/−^* mice Paganini et al., 2019; Kodama et al., 2020	Short stature, thoracic kyphosis, delta phalanx	N.D.	N.D.
	Recessive multiple epiphyseal dysplasia *(MIM: 617719/AR)* Balasubramanian et al., 2017	Mild short stature, joint pain, early-onset osteoarthropathy						
*TGDS*	Catel–Manzke syndrome (CATMANS) *(MIM: 616145/AR)* Ehmke et al., 2014; Pferdehirt et al., 2015; Schoner et al., 2017; Boschann et al., 2020	Pierre Robin sequence, clinodactyly of index finger to a bilateral hyperphalangy		Radial deviation of index fingers due to presence of accessory bones between 2nd metacarpal and proximal phalanx	N.D.	N.D.	N.D.	N.D.
*TMEM165*	TMEM-CDG (*MIM: 614727/AR)* Foulquier et al., 2012; Zeevaert et al., 2013; Schulte Althoff et al., 2016	Post-natal growth retardation and with severe spondylo-, epi-, and metaphyseal skeletal dysplasia and joint laxity	Psychomotor retardation, hypotonia		*WAP-Cre; Tmem165^*fl*/*fl*^* Snyder et al., 2019	Defective milk production	*tmem165* morphant zebrafish Bammens et al., 2015	Reduced size and craniofacial defects

*Gray-shaded rows indicate skeletal dysplasias that belong to CMD group*.

PGs are highly diverse ECM components. Indeed, they can be composed of different core proteins with one or more GAG chain(s) of various subtypes and are subjected to variable degrees of post-translational modifications, including glycosylation and sulfation. Altogether, this leads PG to have a multitude of biological functions. Indeed, PGs promote ECM assembly by interacting with other ECM components, regulate ECM physical properties, and serve as a reservoir for various growth factors (Schaefer and Schaefer, 2010; Iozzo and Schaefer, 2015). In particular, PGs are highly expressed in cartilage ECM and play a major role in chondrocyte maturation and bone formation through endochondral ossification. PGs are also, through their ability to bind and retain water in the matrix, a critical component of articular cartilage, ensuring adequate mechanical properties and integrity maintain of articular cartilage (Martínez-Moreno et al., 2019).

## Chondroitin Sulfate, Dermatan Sulfate, Heparan Sulfate Biosynthesis

**Figure 2:**
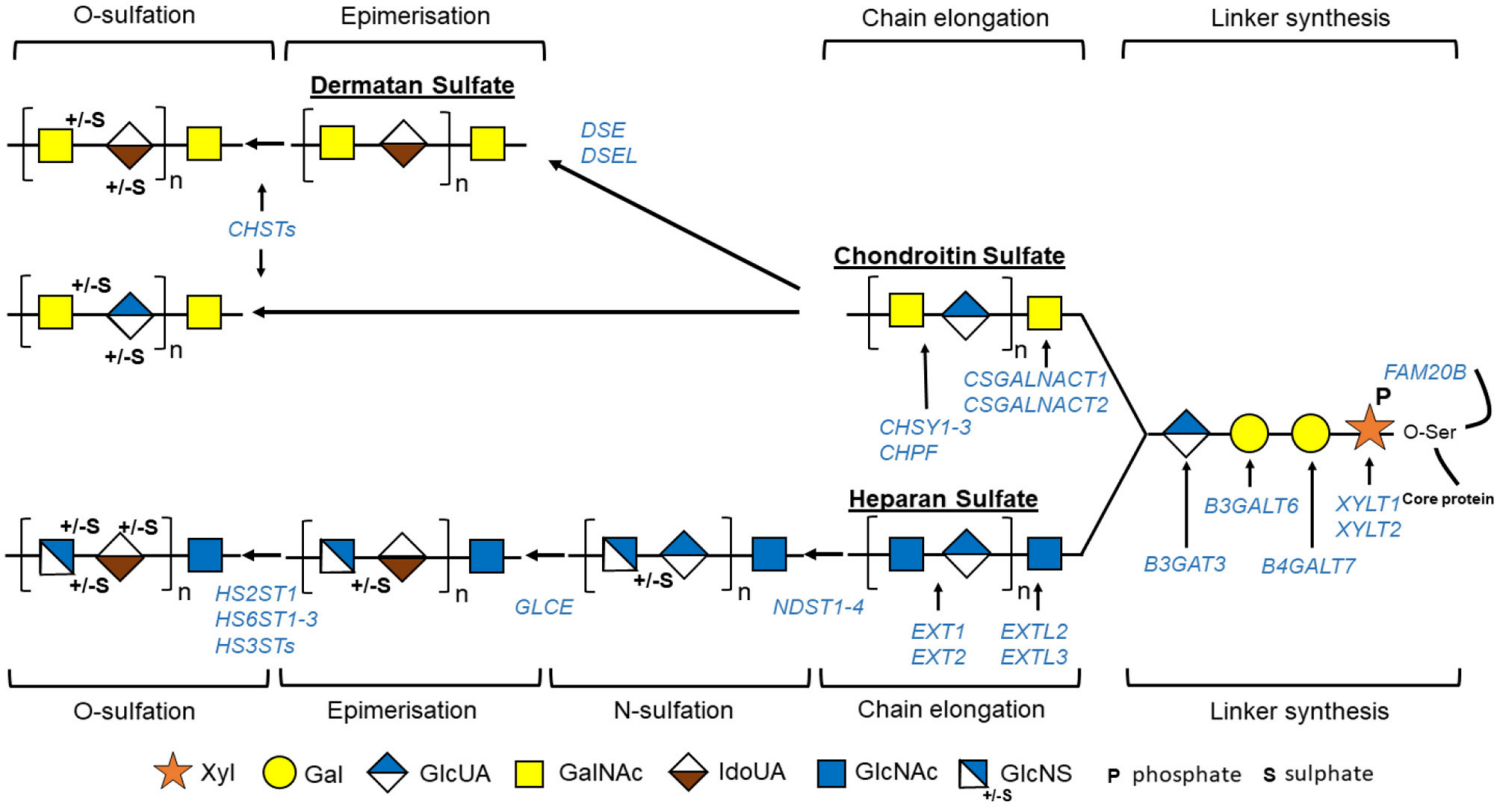
Schematic view of GAG biosynthesis: GAG biosynthesis is initiated by sequential addition to specific serine residues of PG core protein of one xylose (Xyl), two galactoses (Gal), and one glucuronic acid (GlcUA) constituting tetrasaccharide linker region common to tree groups of GAG, i.e., heparan sulfate, chondroitin sulfate, and dermatan sulfate. GAG chains will then be elongated by binding specific repetitive disaccharides [*N*-acetylgalactosamine (GalNAc) and glucuronic acid (GlcUA) for chondroitin sulfate and *N*-acetylglucosamine (GlcNAc) and GlcUA for heparan sulfate]. Some residues are modified: epimerization of GlcUA to iduronic acid (IdoUA) that will generate dermatan sulfate from chondroitin sulfate, or *N*-sulfation/*N*-deacetylation of GlcNAc to *N*-sulfoglucosamine (GlcNS) followed by epimerization of GlcUA to IdoUA for heparan sulfate. Finally, GAGs are further modified by O-sulfation. Different enzymes implicated in these processes are indicated in blue. “HS3TSs” include seven HS3ST (HS3ST1, 2, 3A, 3B, 4, 5, and 6), and “CHSTs” include four chondroitin-4-O-sulfotransferases (CHST11-14), two chondroitin-6-O-sulfotransferases (CHST3 and 7), one GalNAc-4-O-sulfate-6-O-sulfotransferase (CHST15), and one uronyl-2-O-sulfotransferase.

**Figure 3:**
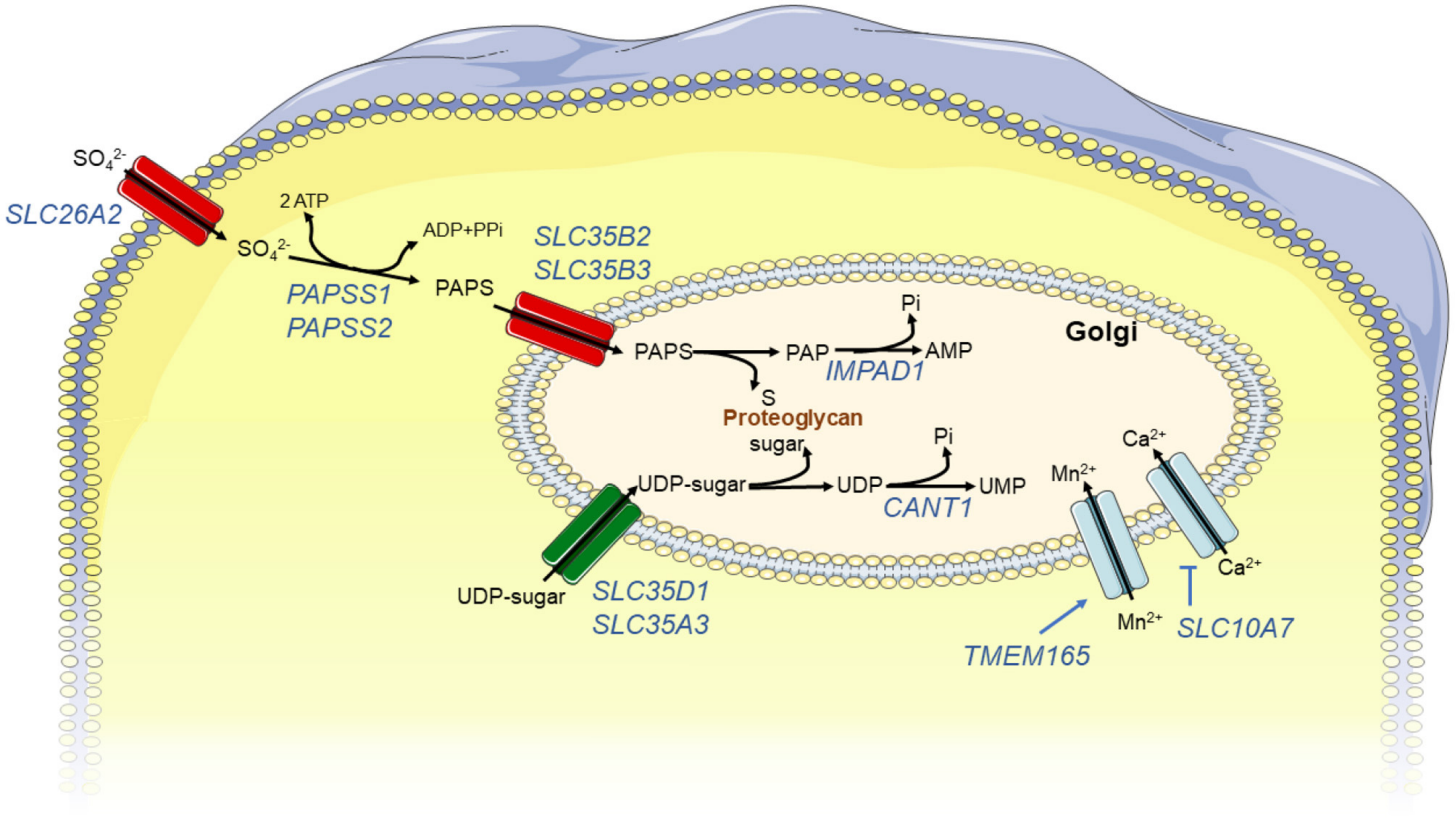
Schematic view of enzymes or transporters associated with GAG synthesis: GAG synthesis requires specific substrates, nucleotide sugars used by glycosyltransferases, and PAPS used by sulfotransferases. Nucleotide sugars and PAPS are synthetized in cytoplasm and are transported in Golgi by specific carriers. Then, glycosyltransferase and sulfotransferase activities produce by-products, UDP and PAP, respectively, that have to be degraded to avoid inhibition of catalytic activity of these enzymes *via* feedback mechanisms. Finally, to assure correct activity of glycosyltransferases, Golgi environment, and in particular its concentration in divalent ions, has to be tightly regulated *via* import/export of these ions. Enzymes, proteins, or transporters implicated in these processes are indicated in blue.

The GAG biosynthesis is a complex process implicating the action of multiple enzymes (Figures 2, 3, Table 2) and that, although it is initiated in the endoplasmic reticulum (ER), occurs mainly in the Golgi apparatus cisternae (Prydz, 2015). GAG biosynthesis is initiated in the ER by the attachment of a xylose (Xyl) residue, using uridine diphosphate (UDP)-Xyl as a donor, to specific serine residues of the freshly synthetized PG core protein by β-xylosyltransferases encoded by *XYLT1* or *XYLT2* (Götting et al., 2007). After Xyl addition and shipment of the xylosylated protein into the Golgi apparatus, a linkage tetrasaccharide is formed by the transfer of two galactose (Gal) residues from UDP-Gal and one GlcUA from UDP-GlcUA *via* the sequential action of β1,4-galactosyltransferase-I (GalT-I), β1,3-galactosyltransferase-II (GalT-II), and β1,3-glucuronosyltransferase-I (GlcAT-I), encoded by *B4GALT7, B3GALT3*, and *B3GAT3* (Okajima et al., 1999; Pedersen et al., 2000; Bai et al., 2001). Some modifications may occur on the linkage tetrasaccharide, including 2-O-phosphorylation of Xyl residue along with sulfation of the first Gal residue at the C-6 position and of the second Gal residue at the C-4 or C-6 position (Gulberti et al., 2005). The phosphorylation, which can be transient, is catalyzed by a GAG-Xyl kinase encoded by *FAM20B* (Koike et al., 2009). This phosphorylation together with sulfation may influence the catalytic activity of GalT-I, GalT-II, and GlcAT-I and thus the linkage region assembly and subsequent GAG elongation (Wen et al., 2014).

**Table 2:** GAG biosynthesis enzymes implicated in defects observed in patient samples.

				**Gene deficiency consequences evidenced in patient samples**	
	**Protein name**	**Function**	**GAG**	**PG**	**Other glycosylation defects**
**Linker**					
*XYLT1*	Xylosyltransferase 1 (*MIM: 608124*)	Transfer of a Xyl residue from UDP-Xyl to specific serine residues of PG core protein Götting et al., 2007	Reduced total GAG synthesis after incubation with methylumbelliferyl-β-D-xylopyranoside in patient fibroblasts Bui et al., 2014	Reduced glycosylation of decorin in patient fibroblast supernatants Schreml et al., 2014	N.D
*XYLT2*	Xylosyltransferase 2 (*MIM: 608125*)	Transfer of a Xyl residue from UDP-Xyl to specific serine residues of PG core protein Götting et al., 2007	Reduced HS staining in patient fibroblasts and reduced CS and HS chains synthesis in patient fibroblasts Munns et al., 2015	N.D.	N.D.
*FAM20B*	Glycosaminoglycan xylosylkinase (*MIM: 611063*)	Phosphorylates initiator xylose residue Koike et al., 2009	N.D.	N.D.	N.D.
*B4GALT7*	Galactosyltransferase I (*MIM: 604327*)	Transfer of first Gal residue to Ser-O-Xyl of tetrasaccharide linkage region Okajima et al., 1999	Reduced epimerization of GAG chain in patient fibroblasts Seidler et al., 2006	Defective biosynthesis of mature decorin and biglycan in patient fibroblasts Quentin et al., 1990; Seidler et al., 2006 Reduced level of bikunin bearing CS chain on Western blot in patient serum Bruneel et al., 2018; Haouari et al., 2020	N.D.
*B3GALT6*	β-1,3-Galactosyltransferase 6 (*MIM: 615291*)	Transfer of second Gal residue to Ser-O-Xyl-Gal of tetrasaccharide linkage region Bai et al., 2001	Reduced HS chains and increased CS and DS chains in patient lymphoblastoid cells Nakajima et al., 2013 Reduced total GAG synthesis in patient fibroblasts Malfait et al., 2013 Disorganized HS GAG ECM in patient fibroblasts Ritelli et al., 2015	Impaired glycanation of decorin in patient fibroblasts Malfait et al., 2013 Reduced level of bikunin bearing CS chain on Western blot in patient serum Bruneel et al., 2018; Haouari et al., 2020 Reduced perlecan immunostaining in patient fibroblasts Ritelli et al., 2015	N.D.
*B3GAT3*	β-1,3-Glucuronyltransferase 3 (*MIM: 606374*)	Transfer of a GlcUA residue to Ser-O-Xyl-Gal-Gal of tetrasaccharide linkage region Pedersen et al., 2000	Reduced CS, DS, and HS chains synthesis in patient fibroblasts Baasanjav et al., 2011	Increased level of DS-free decorin in patient fibroblasts Baasanjav et al., 2011 Reduced level of bikunin bearing CS chain on Western blot in patient serum Bruneel et al., 2018; Haouari et al., 2020	N.D.
**CS/DS chain elongation**					
*CSGALNACT1*	Chondroitin sulfate N-acetylgalactosaminyltransferase 1 (*MIM: 616615*)	Transfer of GalNAc residue onto linker region for initiation of CD/DS synthesis Sato et al., 2003	Reduced number of CS/DS chains in patient fibroblasts Mizumoto et al., 2020	Normal PG synthesis Vodopiutz et al., 2017	N.D.
*CSGALNACT2*	Chondroitin sulfate N-acetylgalactosaminyltransferase 1 (*MIM: 616616*)	CS/DS chain elongation Sato et al., 2003	N.D.	N.D.	N.D.
*CHSY1*	Chondroitin sulfate synthase 1 (*MIM: 608183*)	CS/DS chain elongation Uyama et al., 2002	Decreased CS immunostaining in patient skin Tian et al., 2010	Reduced molecular weight of bikunin bearing CS chain on Western blot in patient serum Bruneel et al., 2018	N.D.
*CHPF*	Chondroitin polymerizing factor (*MIM: 610405*)	CS/DS chain elongation Kitagawa et al., 2001	N.D.	N.D.	N.D.
*CHPF2*	Chondroitin polymerizing factor 2 (*MIM: 608037*)	CS/DS chain elongation Izumikawa et al., 2008	N.D.	N.D.	N.D.
*DSE*	Dermatan sulfate epimerase (*MIM: 605942*)	Epimerization of GlcUA of CS chain into IdoUA converting CS to DS Malmström and Aberg, 1982	Decreased DS disaccharides in patient fibroblasts Müller et al., 2013	Glycanation of decorin is impaired in patient fibroblasts Müller et al., 2013	
*DSEL*	Dermatan sulfate epimerase-like (*MIM: 611125*)	Epimerisation of GlcUA of CS chain into IdoUA converting CS to DS Pacheco et al., 2009	N.D.	N.D.	N.D.
**HS chain elongation**					
*EXTL1*	Exostosin-like glycosyltransferase 1 (*MIM: 601738*)	Transfer of GlcNAc residues to tetrasaccharide linkage region for initiation of HS synthesis Kim et al., 2001	N.D.	N.D.	N.D.
*EXTL2*	Exostosin-like glycosyltransferase 2 (*MIM: 602411*)	Transfer of a GlcNAc residue to a phosphorylated tetrasaccharide linkage region to stop chain elongation Nadanaka et al., 2013b	N.D.	N.D.	N.D.
*EXTL3*	Exostosin-like glycosyltransferase 3 (*MIM: 605744*)	Transfer of a GlcNAc residue to tetrasaccharide linkage region for initiation of HS synthesis Kim et al., 2001	Lower HS concentration in patient fibroblasts, increased CS and DS concentrations in patient serum and urine Oud et al., 2017 Abnormal HS sulfation pattern in patient fibroblasts Volpi et al., 2017	N.D.	N.D.
*EXT1/EXT2*	Exostosin glycosyltransferase 1 (*MIM: 608177*)/Exostosin glycosyltransferase 2 (*MIM: 608210*)	HS polymerization McCormick et al., 2000	Reduced HS/CS ratio in patient serum Anower-E-Khuda et al., 2013	N.D.	N.D.
**Sulfation**					
*SLC26A2*	DTD sulfate transporter (*MIM: 606718*)	Transports sulfate ions across cell membrane Hästbacka et al., 1994	Undersulfated CS in patient fibroblasts and cartilage Rossi et al., 1998	N.D.	N.D.
*PAPSS1*	3'-phosphoadenosine 5'-phosphosulfate synthase (*MIM: 603262*)	Synthetizes universal sulfate donor (PAPS) Xu et al., 2000	N.D.	N.D.	N.D.
*PAPSS2*	3'-phosphoadenosine 5'-phosphosulfate synthase 2 (*MIM: 603005*)	Synthetizes universal sulfate donor (PAPS) Xu et al., 2000	Undersulfation of CS in patient urine Toledo et al., 1978	N.D.	N.D.
*SLC35B2 (PAPST1)*	Solute carrier family 35 (3'-phosphoadenosine 5'-phosphosulfate transporter), member B2 (*MIM: 610788*)	Transports PAPS from cytosol to Golgi Kamiyama et al., 2003	N.D.	N.D.	N.D.
*SLC35B2 (PAPST2)*	Solute carrier family 35 (3'-phosphoadenosine 5'-phosphosulfate transporter), member B2 (*MIM: 610845*)	Transports PAPS from cytosol to Golgi Kamiyama et al., 2006	N.D.	N.D.	N.D.
*CHST3*	Carbohydrate sulfotransferase 3 (*MIM: 603799*)	Transfers sulfate from PAPS to C6 of GalNAc residues of CS Tsutsumi et al., 1998	Reduction of 6-O-sulfated disaccharide in patient fibroblasts and urine Thiele et al., 2004. Increase of 4-O-sulfated disaccharide in patient fibroblasts Hermanns et al., 2008	N.D.	N.D.
*CHST11*	Carbohydrate sulfotransferase 11 (*MIM: 610128*)	Transfers sulfate from PAPS to GalNAc residues of DS Hiraoka et al., 2000	N.D.	N.D.	N.D.
*CHST14*	Carbohydrate sulfotransferase 14 (*MIM: 608429*)	Transfers sulfate from PAPS to C4 of GalNAc residues of CS Evers et al., 2001	Reduced DS biosynthesis and increased CS concentration in patient fibroblasts Dündar et al., 2009; Miyake et al., 2010 DS not detected in patient urines Mizumoto et al., 2017	Decorin depleted of DS chains, replaced by CS chains in patient fibroblasts Miyake et al., 2010	N.D.
*IMPAD1*	Inositol monophosphate domain-containing protein 1 (*MIM: 614010*)	Hydrolyses by-product of sulfotransferase reactions, PAP, in AMP and phosphate Frederick et al., 2008	N.D.	N.D.	N.D.
**Transporter or other**					
*SLC35D1*	Solute carrier family 35 (UDP-glucuronic acid/UDP-N-acetylgalactosamine dual transporter), member D1 (*MIM: 610804*)	Transports UDP-GlcUA/UDP-GalNAc from cytosol to Golgi Muraoka et al., 2001	N.D.	N.D.	N.D.
*SLC35A3*	Solute carrier family 35 (UDP-N-acetyl glucosamine transporter), member 3 (*MIM: 605632*)	Transports UDP-GlcNAc from cytosol to Golgi Maszczak-Seneczko et al., 2013	N.D.	Reduced molecular weight of bikunin bearing CS chain on Western blot in patient serum Haouari et al., 2020	Reduced N-glycan branching in patient cells and plasma Edvardson et al., 2013
*SLC10A7*	Solute carrier family 10 (sodium:bile acid cotransporter family), member 7 (*MIM: 611459*)	Negative regulator of intracellular calcium homeostasis Karakus et al., 2020	Significant reduction of HS proportion linked to increased CS proportion in patient fibroblasts Dubail et al., 2018	N.D.	Defective N glycosylation in patient serum Ashikov et al., 2018; Dubail et al., 2018
*CANT1*	Calcium-activated nucleotidase 1 (*MIM: 613165*)	Hydrolyses UDP to UMP and phosphate in Golgi Smith et al., 2002	Reduced GAG synthesis after stimulation by β-D-xyloside in patient fibroblasts Nizon et al., 2012b	N.D.	N.D.
*TGDS*	TDP-glucose 4,6-dehydratase (*MIM: 616146*)	cTDP-D-glucose 4,6-dehydrogenase homologous to a UDP-glucuronate decarboxylase 1 that catalyzes synthesis of UDP-xylose from UDP-glucuronate Ehmke et al., 2014	N.D.	N.D.	N.D.
*TMEM165*	Transmembrane protein 165 (*MIM: 614726*)	Putative role of Mn2+ transporter Dulary et al., 2017	N.D.	Reduced molecular weight of bikunin bearing CS chain on Western blot in patient serum Haouari et al., 2020	Increased undersialylated and undergalactosylated glycans in patient serum Foulquier et al., 2012; Xia et al., 2013; Zeevaert et al., 2013

Once the linkage region is completed, two types of reactions occur and determine the type of GAG being synthetized: addition to the linkage tetrasaccharide of either a β4-linked GalNAc, which will initiate CS/DS assembly, or an α4-linked GlcNAc, which will initiate HS assembly (Lindahl et al., 2017).

In CS, the process starts with the transfer of a GalNAc from UDP-GalNAc to the last GlcUA residue of the linkage region by specific β1,4-*N*-acetlygalactosaminyltransferases, CSGalNAcT1 and CSGalNAcT2 (encoded by CSGALNACT1 and CSGALNACT2, respectively) (Uyama et al., 2002; Sato et al., 2011). CS chains are then polymerized by the action of one or more enzymes having both β3 glucuronosyltransferase and β4 *N*-acetylgalactosaminyltransferase activities, the chondroitin synthases (CHSY1-3) (Kitagawa et al., 2001). During this step, the chondroitin polymerizing factor, lacking independent activity, will interact with the chondroitin synthases and enhance the CS elongation (Izumikawa et al., 2008). CSs are then subjected to modifications such as epimerization and sulfation throughout the GAG synthesis process or just before PG secretion (Lindahl et al., 2017). CS sulfation in an elaborate process involved multiple sulfotransferases, three chondroitin-4-O-sulfotransferases (CHST11-13) for C4 sulfation of GalNac residues, two chondroitin-6-O-sulfation (CHST3 and 7) for C6 sulfation of GalNac residues, one GalNac-4-O-sulfate-6-O-sulfotransferase (CHST15) for sulfation of disulfated GalNAc, and one uronosyl-2-O-sulfotransferase for C2 sulfation of GlcUA (Mizumoto et al., 2013).

DSs are generated from CS by C5 epimerization of GlcUA to IdoA by two DS epimerases (DSE1–2) and sulfation in distinct positions by dermatan-4-O-sulfotransferase (CHST14) and uronosyl-2-O-sulfotransferase (Malmström and Aberg, 1982).

In HS, the assembly is initiated by the addition of a GlcNAc residue by an α1,4-*N*-acetylglucosaminyltransferase-I (GlcNAcT-I) encoded by *EXTL3* (Kim et al., 2001). HS further elongation is carried out by HS polymerase complex formed by two enzymes with N-acetylglucosaminyltransferase and glucuronyltransferase activities and encoded by *EXT1* and *EXT2* (McCormick et al., 2000). HS then undergoes extensive modification reaction creating clusters of sulfated domains interspersed with an unsulfated region (Esko and Selleck, 2002). Those modifications are initiated by *N*-deacetylase/*N*-sulfotransferases (NDST1–4), which induce the N-sulfation of 40–50% of GlcNAc to N-sulfoglucosamine (GlcNS) followed by conversion of adjacent GlcA to idorunate (IdoA) by a glucuronyl epimerase. O-sulfotransferases can then modify these GlcNS/IdoA rich domains. HS2ST1 catalyzes the 2-O-sulfation of IdoA residues. IdoA(2S)-GlcNS can then be further modified by the addition of 6-O-sulfate and less frequently by addition of 3-0-sulfate groups to the GlcNS residues, by the action of 6-O-sulfotransferases (HS6ST1-3) and seven 3-O-sulfotransferases (HS3ST), respectively.

The sulfation of GAG is a crucial process during PG synthesis and is required for GAG physiological functions. In the Golgi apparatus, sulfotransferases use the 3′-phosphoadenosine 5′-phosphosulfate (PAPS) as a universal sulfate donor to transfer sulfate to specific residues of GAG chains (Paganini et al., 2020). PAPS is synthetized in the cytosol from adenosine triphosphate (ATP) and inorganic sulfate. The latter is transported from the extracellular environment into the cells through a sulfate/chloride antiporter named SLC26A2 (Hästbacka et al., 1994). PAPS synthesis takes place in two sequential steps by the action of a bifunctional enzyme, the PAPS synthase (Xu et al., 2000). The ATP sulfurylase activity firstly catalyzes the production of adenosine 5′-phosphosulfate (APS) from sulfate and ATP; subsequently, APS kinase activity produces PAPS from APS and ATP. Once PAPS is synthetized in the cytosol, it is translocated into the Golgi by two specific PAPS transporters (PAPS transporters 1 and 2) (Kamiyama et al., 2003, 2006). As a consequence of sulfotransferase activity, PAP is released and can inhibit those sulfotransferases *via* negative feedback. To prevent this, PAP is rapidly degraded into adenosine monophosphate and phosphate by a Golgi resident adenosine 3′, 5′-biphosphate 3′-phosphatase (gPAPP), encoded by *IMPAD1* (also known as *BPNT2*) (Frederick et al., 2008).

Nucleotide sugars such as PAPS are synthetized in the cytoplasm and have to be transported into the Golgi by specific carriers, such as SLC35D1 or SLC35A3, that by an antiport mechanism will export nucleoside monophosphates in the cytosol (Muraoka et al., 2001; Maszczak-Seneczko et al., 2013). This nucleotide sugar import seems to be a rate-limiting step as increased UDP-N-acetylhexosamine availability leads to enhancement of the incorporation into glycoconjugates (Pels Rijcken et al., 1995).

GAG elongation and modification reactions probably colocalized within the Golgi cisternae, most likely by the formation of supramolecular complexes that coordinate these reactions. A correct conformation of Golgi cisternae and organization of their enzymatic content, as well as an adequate Golgi environment, i.e., a properly established pH gradient and concentration of ions such as Ca2+, are also required for correct GAG formation (Prydz, 2015). Any disturbances of this chain of reactions will lead to the incapacity of a cell to construct correct glycanic chains.

## Chondrodysplasia With Multiple Dislocation and Associated Animal Models

So far, up to 27 distinct human genetic disorders have been associated with pathogenic variants in 23 genes encoding proteins implicated in GAG biosynthesis (Table 1, Figures 2, 3). With few exceptions, such as *EXT1*/*EXT2* or *SLC35D1*, the vast majority of the pathological variants identified in these genes are responsible for skeletal dysplasia associating short stature and joint laxity and/or large joint dislocations, characteristic of the CMD group (Table 1, gray rows). Clinical features of inborn errors of GAG biosynthesis, as well as the phenotype of existing related deficient animal models, are described in Table 1. The functional consequences of these inborn errors on GAG or PG synthesis, evidenced in patient samples, are listed in Table 2.

In the following section, we will focus on genes implicated in CMD.

### Defective Linker Region Biosynthesis

Disorders due to mutations in *XYLT1, B4GALT7, B3GALT6*, and *B3GAT3*, encoding enzymes involved in the synthesis of the common linker region, are now frequently referred to as “linkeropathies.” *FAM20B*, which encodes a xylose kinase, in which its activity affects the synthesis of the common linker region, will also be described in this section.

#### XYLT1

Desbuquois dysplasia type 2 (DD 2), called Baratella–Scott syndrome, is caused by homozygous mutations in *XYLT1* (Bui et al., 2014). DD 2 is characterized by severe pre- and post-natal growth retardation, dislocation of large joints with generalized joint laxity, short, long bones with a monkey wrench appearance to the proximal femur, advanced ossification of carpal and tarsal bones, and facial dysmorphisms, including flat face with prominent eyes. It is also associated with alterations in the fat distribution, variable degree of intellectual disabilities, and cleft palate. Reduced total GAG synthesis and decorin glycosylation were detected in fibroblasts of affected individuals compared with healthy controls (Bui et al., 2014; Schreml et al., 2014). Before the identification of pathogenic variants in humans, a mutant mouse and a mutant zebrafish were described, both exhibiting skeletal alterations. In *pug* mice, identified by ENU mutagenesis screen, XYLT1 deficiency due to homozygous missense mutation in *Xylt1* is responsible for disproportionate dwarfism due to an early chondrocyte maturation and early ossification (Mis et al., 2014). A mutagenesis screen in zebrafish, isolating mutant fish harboring decreased cartilage matrix and increased perichondral bone, leads to the generation of *xylt1* mutants (Eames et al., 2011). These mutant zebrafish failed to produce wild-type levels of CS and exhibited altered craniofacial skeletal morphology.

#### B4GALT7

Biallelic variants in *B4GALT7* cause Ehlers–Danlos syndrome (EDS) progeroid type 1, now called EDS spondylodysplatic type 1 characterized by hypermobile joints, an aged appearance with loose yet elastic skin, poor wound healing, hypotonic muscles, craniofacial dysmorphism, short stature, developmental delays, and generalized osteopenia (Okajima et al., 1999). Homozygous mutations in *B4GALT7* are also responsible for a variant of Larsen syndrome frequent on the La Reunion Island, called Larsen of Reunion Island syndrome, which has clinical manifestations including characteristic facial features, multiple dislocations, short stature, and hyperlaxity (Cartault et al., 2015). In patient fibroblasts, B4GALT7 mutations result in abnormal biosynthesis of mature decorin and biglycan with reduced GAG chain epimerization (Quentin et al., 1990; Seidler et al., 2006). Recently, a reduced level of bikunin bearing CS chain was detected by Western blot in patient serum compared with healthy controls (Bruneel et al., 2018; Haouari et al., 2020). Both knockdown (morphant) and mosaic knockdown *b4galt7* zebrafish models presented short stature, deformed pectoral fins, craniofacial dysmorphism, and reduced mineralization (Delbaere et al., 2020).

#### B3GALT6

Mutations in *B3GALT6* cause EDS progeroid type 2, also called EDS spondylodysplastic type 2 and spondyloepimetaphyseal dysplasia with joint laxity, Beighton type (Vorster et al., 2015). The main clinical features for this autosomal recessive syndrome include an aged appearance with loose but elastic skin and defective wound healing, hypermobile joints, developmental delay, short stature, craniofacial disproportion, kyphoscoliosis, epimetaphyseal dysplasia, generalized osteopenia, and hypotonic muscles. Fibroblasts from affected individuals exhibited altered GAG synthesis with impaired glycanation of decorin and marked reduction of HS synthesis (Malfait et al., 2013; Nakajima et al., 2013; Ritelli et al., 2015). As for patients with *B4GALT7* mutations, a reduced level of bikunin bearing CS chain was detected by Western blot in patient serum compared to healthy controls (Bruneel et al., 2018; Haouari et al., 2020).

#### B3GAT3

Recessive variants in *B3GAT3* cause Larsen-like syndrome characterized by short stature, multiple joint dislocations, scoliosis, osteopenia, and cranial dysmorphisms such as a flattened midface, hypertelorism, depressed nasal bridge, and prominent forehead (Van Damme et al., 2018). Congenital heart defects, including mitral valve prolapse, ventricular defect, and bicuspid aortic valve, can be observed in those patients. Patient fibroblasts exhibited reduced CS, DS, and HS and an increased level of DS-free decorin as compared with healthy controls (Baasanjav et al., 2011). Moreover, a reduced level of bikunin bearing CS chain was detected in patient serum compared with healthy controls (Bruneel et al., 2018; Haouari et al., 2020). *B3gat3*-deficient mice synthesized a smaller CS and HS chain in their blastocysts than that of heterozygous mice and exhibited an embryonic lethality before the eight-cell stage due to the failure of cytokinesis (Izumikawa et al., 2010). On the other hand, b3gat3 mutant zebrafish presented with abolished CS synthesis and abnormal pharyngeal cartilage morphogenesis (Holmborn et al., 2012).

#### FAM20B

Recently, compound heterozygous mutations in *FAM20B* have been described in patients with a lethal form of neonatal short-limb dysplasia characterized by very short stature and multiple dislocations of the large joints, thoracic hypoplasia, respiratory failure, and midface hypoplasia (Kuroda et al., 2019). *Fam20b*-deficient mice exhibited embryonic lethality at embryonic day 13.5 with multiorgan hypoplasia (Vogel et al., 2012). Furthermore, inactivation of *Fam20b* in several murine-specific tissues or cell types leading to skeletal defects demonstrated a role of *Fam20b* in bone development (Tian et al., 2015; Ma et al., 2016, 20; Liu et al., 2018; Saiyin et al., 2019). Similar to *xylt1* zebrafish mutant, *fam20b* zebrafish mutant exhibited altered craniofacial skeletal morphology, decreased cartilage matrix, and increased perichondral bone (Eames et al., 2011).

### Defective Glycosaminoglycan Chain Elongation or Epimerization

CSGALNACT1, CHSY1, and DSE Encode for enzymes implicated in CS/DS chain elongation and epimerization of CS to DS. No joint laxity or joint dislocations were described in disorders linked to pathogenic variants encoding for enzymes implicated in HS chains elongation. They will thus not be discussed in this section.

#### CSGALNACT1

Pathogenic variants in CSGALNACT1 have been identified recently in patients with a skeletal dysplasia characterized by a mild micromelic and non-proportioned stature, joint laxity, and advanced bone age (Vodopiutz et al., 2017; Mizumoto et al., 2020). Altered levels of CS, DS, and HS moieties were observed in patient fibroblasts compared with healthy controls (Vodopiutz et al., 2017; Mizumoto et al., 2020). *CSGalNAcT1*-deficient mice were described several years before and presented with slight dwarfism and abnormalities in perineural nets and behavior (Watanabe et al., 2010; Sato et al., 2011; Yoshioka et al., 2017).

#### CHSY1

Temtamy preaxial brachydactyly syndrome is caused by homozygous mutations in *CHSY1* (Li et al., 2010). It is characterized by bilateral and symmetric preaxial brachydactyly and hyperphalangism of digits, growth retardation, facial dysmorphism, deafness, and delayed motor and mental development. Patient skin biopsy exhibited decreased CS-specific immunostaining compared with controls, and reduced molecular weight bikunin bearing CS chain was detected by Western blot in patient serum compared with healthy controls (Tian et al., 2010; Bruneel et al., 2018). *Chsy1*-deficient mice developed a phenotype mimicking the human pathology, presenting with a chondrodysplasia and decreased bone density with severe digit patterning defects (Wilson et al., 2012). In the same way, *chsy* morphant zebrafish presented with reduced body length, compromised pectoral fin formation, cranial dysmorphism, and inner ear formation defects (Li et al., 2010).

#### DSE

Homozygous mutations in DSE cause EDS musculocontractural type 2 characterized by joint hypermobility (finger, elbow, and knee), distinctive facial features, multiple congenital contracture contractures (thumbs and feet), and myopathy (Müller et al., 2013). Additional features might include cardiac, valvular, respiratory, gastrointestinal, and ophthalmic complications. Decreased DS level and reduced glycanation of decorin have been evidenced in patient fibroblasts compared with controls (Müller et al., 2013). *Dse*-deficient mice were smaller, with a kinky tail at birth, altered skin morphology and skin tensile strength, and abdominal wall defects (Maccarana et al., 2009; Gustafsson et al., 2014). On the other hand, *dse* morphant xenopus showed abnormal development of neural crest-derived structure (Gouignard et al., 2016).

### Defective Glycosaminoglycan Sulfation

SLC26A2, CHST3, CHST11, CHST14, and IMPAD1 Encode for transporter and enzymes implicated in sulfation CS/DS chains. Except from a very recent manuscript demonstrating that mutations in *HS2ST1* are responsible for a syndrome characterized by developmental delay with corpus callum, skeletal, and renal abnormalities (Schneeberger et al., 2020), pathogenic variants in enzymes implicated in HS sulfation have not been associated with skeletal dysplasia and will not be described here.

#### SLC26A2

*SLC26A2*-linked chondrodysplasias form a heterogeneous group of four different skeletal diseases caused by mutations in *SLC26A2* (Bonafé et al., 1993a,b,c; Superti-Furga and Unger, 1993). They include decreasing order of severity, from lethal to mild, achondrogenesis type 1B, atelosteogenesis type 2, diastrophic dysplasia (DTD), and recessive multiple epiphyseal dysplasia. Among them, DTD is characterized by short stature, joint deformities and joint contractures, club foot, progressive kyphoscoliosis of the spine, hitchhiker thumb, characteristic ear deformities, and, occasionally, cleft palate. Undersulfated CSs were detected in patient fibroblasts and cartilage biopsies (Rossi et al., 1998). The skeletal phenotype of *dtd* mice, an animal model of human DTD, included reduced skeletal growth with long bone deformities and osteoporosis. Moreover, growth plate cartilage showed reduced toluidine blue staining, chondrocytes of irregular size, and delayed secondary ossification center formation (Forlino et al., 2005). Slc26a2 morphant zebrafish exhibited, on the other hand, an abnormal otic development (Liu et al., 2015).

#### CHST3 (CST6)

Loss-of-function mutations in *CHST3* cause spondyloepiphyseal dysplasia with congenital joint dislocations also called recessive Larsen syndrome (Thiele et al., 2004). It is characterized by short stature of prenatal onset, large joint dislocations at birth (knees and/or hips), elbow joint dysplasia with subluxation and limited extension, clubfoot, and progressive kyphosis appearing during late childhood. Sulfation defects were detected in patient fibroblasts with a reduction of 6-O-sulfated disaccharides and increased 4-O-sulfated disaccharides compared with controls (Hermanns et al., 2008). Furthermore, a reduction in 6-O-sulfated disaccharides in patient urine was reported (Thiele et al., 2004). *Cst6*-deficient mice did not develop skeletal dysplasia but exhibited a decreased number of naïve T lymphocytes in the spleen (Uchimura et al., 2002).

#### CHST11 (C4ST1)

Mutations in *CHST11* have been recently identified in patients with osteochondrodysplasia, brachydactyly, and overlapping malformed digits (Shabbir et al., 2018). Individuals with *CHST11* mutations have bilateral symmetric skeletal defects affecting primarily the limbs with shortening of the lower leg bones leading to mild short stature, associated with hand and foot malformations, predominantly brachydactyly and overlapping digits. Scoliosis, dislocated patellae and fibulae, and pectus excavatum can also be observed. *C4st1* mutant mice, previously generated by gene trap mutagenesis, exhibited a severe chondrodysplasia linked to abnormalities in the long bone growth plate (Klüppel et al., 2005). *C4st1* mutant embryos developed several skeletal malformations, including a small rib cage, very short limbs, a twisted vertebral column, and a dome-shaped skull.

#### CHST14

Mutations in *CHST14* cause EDS musculocontractural type 1 that has a similar clinical phenotype to EDS muscolocontractural type 2 due to mutations in CHST3 (Malfait et al., 2010). It is characterized by typical facial appearance, thumb and finger congenital contractures, clubfeet, joint hypermobility, severe kyphoscoliosis, muscular hypotonia, ocular involvement, and characteristic cutaneous features including skin hyperextensibility, thin skin, easy bruisability, atrophic scar, and increased palmar winkling. Decreased dermatan sulfate and increased chondroitin sulfate chain synthesis was measured in patient fibroblasts compared with control, and although DS could be detected in urine from healthy controls, it was not the case in urine from patients with *CHST14* mutations (Dündar et al., 2009; Miyake et al., 2010; Mizumoto et al., 2017). *Chst14*-deficient mice had a smaller body mass, a kinked tail, reduced fertility, and a more fragile skin than wild-type mice (Akyüz et al., 2013; Hirose et al., 2019).

#### IMPAD1

Mutations in *IMPAD1* cause chondrodysplasia with joint dislocations, gPAPP type (Vissers et al., 2011; Nizon et al., 2012a). It is characterized by chondrodysplasia with severe growth retardation with brachydactyly, joint dislocation, and cleft palate with micrognathia. Radiographs of hands and feet revealed abnormal extremities with the presence of many accessory bones, abnormally shaped phalanges, and carpal synostosis. *Impad1*-deficient mice developed a perinatal lethal phenotype with severe dwarfism, skeletal defects, and abnormal joint formation (Sohaskey et al., 2008).

### Defective Activity of Transporters and Other Golgi Proteins

In this section, we focus on genes encoding transporter or protein expressed in the Golgi for which pathogenic variants have been identified in patients with CMD and associated with defects in GAG biosynthesis, even if their exact functions on GAG biosynthesis have not been elucidated.

#### SLC35A3

Pathogenic variants in *SLC35A3* have been identified in patients with epilepsy, mental retardation, and multiple skeletal defects, including shortened long bones, vertebral anomalies, large joint dislocation, and arthrogryposis (Edmondson et al., 2017; Marini et al., 2017). The skeletal features are similar to those observed in the complex vertebral malformation phenotype observed in cattle and due to homozygous missense mutation in bovine *Slc35a3* (Thomsen et al., 2006). SLC35A3 encodes a carrier that transports UDP-GlcNAc from the cytosol to the Golgi, where it serves as a substrate for glycosyltransferases (Maszczak-Seneczko et al., 2013). Reduced N-glycan branching has been evidenced in patient cells and plasma compared with control (Edvardson et al., 2013). Moreover, due to its localization and the transporter substrate, it was assumed that SLC35A3 might affect GAG metabolism, and, recently, an abnormal migration profile of bikunin bearing CS chain was observed on Western blot on patient serum (Haouari et al., 2020).

#### SLC10A7

Biallelic mutations in *SLC10A7* have been identified in skeletal dysplasia with amelogenesis imperfecta characterized by a pre- and post-natal short stature, large joint dislocations, luxation of knees with genua valga, hypomineralized amelogenesis imperfecta, decreased bone density, “monkey wrench” appearance of the proximal femora, small epiphyses, advanced carpal ossification abnormal vertebrae, hyperlordosis or kyphoscoliosis, and dysmorphic facial features including Pierre–Robin sequence, micrognathia, and flat face (Ashikov et al., 2018; Dubail et al., 2018). Additional features included heart defects, hearing loss, and obesity. *SLC10A7* codes for 10-transmembrane-domain transporter of unknown substrate specificity located at the Golgi and plasma membrane. Although its function remains unknown, it has been demonstrated that it negatively affects intracellular calcium homeostasis and increased calcium intake that have been measured in patient fibroblasts compared with controls (Dubail et al., 2018; Karakus et al., 2020). This inadequate intracellular calcium influx most likely disturbs the Golgi ionic environment and in fine glycosyltransferase activities. Significant reduction of HS proportion linked to increased CS proportion was observed in patient fibroblasts compared with control (Dubail et al., 2018). Interestingly, defective N-glycosylation was also detected in patient serum (Ashikov et al., 2018; Dubail et al., 2018). *Slc10a7*-deficient mice recapitulated human phenotype with short stature, growth plate disorganization, low bone density, and tooth enamel defects (Dubail et al., 2018). Moreover, alterations of HS/CS content similar to those measured in patient fibroblasts were detected in *Slc10a7*-deficient mouse cartilage. On the other hand, slc10a7 morphant zebrafish presented with abnormal development of several cartilage elements and a strong reduction in bone mineralization (Ashikov et al., 2018).

#### CANT1

Mutations in CANT1 cause Desbuquois dysplasia type 1 characterized by severe pre-natal and post-natal growth retardation with short extremities, joint laxity, and progressive scoliosis (Huber et al., 2009). The main radiologic features include short, long bones with metaphyseal flaring, a “monkey wrench” appearance of the femur neck, and advanced carpal and tarsal ossification with the presence of an extra ossification center, a delta phalanx, between the proximal phalanx of the index and bifid distal thumb phalanx. A milder variant of Desbuquois dysplasia type 1, referred to as “Kim variant,” with hands appearing almost normal externally, but that on radiographic analyses are characterized by elongated phalanges, short metacarpals, and remarkably advanced carpal bone age, has also been linked to pathogenic variants in *CANT1* (Furuichi et al., 2011). *CANT1* codes for calcium-activated nucleotidase, an ER and Golgi nucleotidase that hydrolyses UDP to UMP and phosphate (Smith et al., 2002). Although it has been suggested that CANT1 deficiency would lead to inhibition of glycosyltransferase activities and reduced transport in the Golgi of UDP-sugar *via* negative feedback resulting from increased Golgi UDP level and that CANT1 is implicated, through inositol 1,4,5-triphosphate receptor activation, in vesicular trafficking in Golgi cisternae by calcium release, both potentially affecting GAG synthesis, its exact function remains unknown (Huber et al., 2009; Nizon et al., 2012b). Demonstrating the implication of CANT1 in GAG biosynthesis regulation, reduced GAG synthesis after stimulation by β-D-xyloside has been measured in patient fibroblasts compared with controls (Nizon et al., 2012b). *Cant1*-deficient mice recapitulated the human phenotype of patients with Desbuquois dysplasia type 1, with short stature, thoracic kyphosis, and delta phalanx (Paganini et al., 2019). *Cant1* deficiency led to altered GAG synthesis, with reduced chain length and increased sulfation and delayed secretion of PG in the ECM.

#### TMEM165

Congenital disorder of glycosylation (CDG), type IIk or TMEM165-CDG, is caused by biallelic mutations in TMEM165 (Foulquier et al., 2012). The most severe phenotypes observed in patients with TMEM165-CDG present growth retardation resistant to human growth hormone, associated with a psychomotor disability, microcephaly, facial hypoplasia, hypotonia, seizures, and hepatosplenomegaly with increased serum transaminases. Skeletal features of these patients include severe dwarfism, osteoporosis, epi-, meta-, and diaphyseal dysplasia, and joint laxity. *TMEM165* encodes the transmembrane protein 165, TMEM165, located in the Golgi membrane and, in a lower proportion, at the plasma membrane, and in late endosomes/lysosomes, and which exact function remains unknown. However, several studies are in favor of a role of TMEM165 in Mn2+ homeostasis in the Golgi, suggesting that TMEM165 could act as a putative Mn2+ transporter. This function on Mn2+ homeostasis is essential for appropriate protein N-glycosylation occurring in the Golgi (Dulary et al., 2017). *TMEM165* deficiency has mostly been associated with *N-, O*-glycosylation defects, with increased undersialylated and undergalactosylated glycans, as well as in high mannose type N-Glycan detected in patient serum compared with healthy control (Foulquier et al., 2012; Xia et al., 2013; Zeevaert et al., 2013). However, recently, an abnormal migration profile of bikunin bearing CS chain was observed on a Western blot on patient serum (Haouari et al., 2020). Moreover, tmem165 deficiency in zebrafish led to alterations in CS PG expression detected by immunohistochemical staining of cartilage (Bammens et al., 2015). Altogether, those results imply that TMEM165 deficiency can also lead to GAG synthesis defects. Analyses of these t*mem165* morphant zebrafish demonstrated skeletal abnormalities, more particularly in craniofacial structures with altered bone growth and development. Complete *Tmem165* deficiency in mice has not yet been described, but conditional deletion of *Tmem165* in the mammary gland led to abnormal milk production due to defective lactose biosynthesis (Snyder et al., 2019).

## Glycosaminoglycan Synthesis-Related Disorders Form a Subgroup of Congenital Disorders of Glycosylation

CDG are an expanding group of rare, multisystem, underdiagnosed heterogeneous diseases caused by deficient or improper synthesis or attachment of glycans to proteins and lipids. More than 130 inherited disorders have been identified so far, most of them following an autosomal recessive inheritance pattern (Ondruskova et al., 2021).

Glycosylation is a very important post-translational modification, as it is estimated that at least 2% of the human genome codes for proteins involved in this vital biochemical pathway (Ng and Freeze, 2018). Due to the involvement of glycosylation in various cellular processes, CDG are characterized by multiorgan dysfunction and variable clinical phenotype. Therefore, CDG result in a broad spectrum of pathologies, including skin laxity, skeletal dysplasia, congenital heart defects, neurodevelopmental disorders, and endocrine abnormalities. The most common clinical manifestations include developmental delay, failure to thrive, microcephaly, coagulopathy, and abnormal brain magnetic resonance imaging, including cerebral and/or cerebellar atrophy, cell migration abnormalities, and immune dysfunction (Francisco et al., 2019).

CDG are classified into two groups, CDG I and CDG II. CDG I are defects in the glycan assembly and in the attachment of glycans to proteins in the ER. CDG II are defects in the processing of the already assembled glycans in the Golgi apparatus resulting in truncated and abnormal glycan structures. As it is now considered that glycosylation processes occurring in the ER are required for good folding and stability of the glycoprotein, whereas glycan remodeling in the Golgi will finely regulate the functionality of the protein, CDG I and CDG II will differently affect glycoprotein functions (Freeze, 2007).

Most protein glycosylation disorders are due to defects in the N-glycosylation pathways, but they can also be due to defective O-linked glycans (O-Mannose, O-Glucose, O-Fucose, O-GlcNac, and O-GalNac), GAG, glycosylphosphatidylinositol, and glycolipids (Freeze et al., 2014). As at the Golgi level, the different glycosylation processes coexist, alterations of the Golgi environment/organization, and inadequate supply in common subtrates, will lead to combinations of two or more affected glycosylation pathways. This is well-illustrated by CMD due to mutations in genes coding for transporters or Golgi proteins, i.e., *SLC35A3, SLC10A7*, and *TMEM165*, for which alterations in both N-glycosylation and GAG biosynthesis were detected (Foulquier et al., 2012; Edvardson et al., 2013; Bruneel et al., 2018; Dubail et al., 2018).

## Diagnosis

The group of CMD includes several complex syndromes with overlapping features and other clinical signs that are often non-specific, such as intellectual disability or cardiac defects. Even if few features are typical for some disorders (Table 1), characteristic hand anomalies in *CANT1, IMPAD1, CHSY1*, and *TGDS*-related conditions or amelogenesis imperfecta in dysplasia due to mutations in *SLC10A7*, for example, may help clinicians to orientate the clinical diagnosis. For the vast majority of CMD, phenotype–genotype correlations are still incomplete. Molecular or enzymatic assays to detect specific defects are thus crucial for final diagnostic. However, simple and affordable laboratory tests for use in screening are still missing, and, currently, clinicians must rely on research laboratories to perform the clinical molecular and biochemical tests to confirm the diagnosis. Many research teams have developed and used various techniques to evaluate quantitatively and qualitatively GAG synthesis. With these techniques, they were able to demonstrate in patient biological samples, for some PGs, a reduction of expression, concentration, or molecular weight or an alteration in the concentration of specific disaccharides, sulfated or unsulfated, constituting the GAG (Table 2). In most cases, analyses were performed on lysates or culture media from fibroblasts, cultured or not with radiolabeled substrates, after separation by high-performance liquid chromatography or gel electrophoresis (sodium dodecyl sulfate–polyacrylamide gel electrophoresis). Alternatively, altered migration of some low molecular weight PG was detected by sodium dodecyl sulfate–polyacrylamide gel electrophoresis, and immunoblotting or abnormal GAG expression was revealed by immunohistochemistry or immunofluorescence techniques using primary antibodies against specific GAG residues. Being more easily obtained than fibroblasts, blood and urine samples are more convenient for biochemical testing to perform diagnosis in clinics. Only a few studies have studied the GAG in blood or urine from patients using high-performance liquid chromatography. Recently, abnormal electrophoretic migration profiles of bikunin, a serum PG with a single CS chain, were detected after immunoblotting in serum from patients with specific CMD and, more specifically, from patients with “linkeropathies,” indicating that bikunin is a potential biomarker, easily detectable, for these pathologies (Bruneel et al., 2018; Haouari et al., 2020). Moreover, for patients with mutations in *SLC35A3, SLC10A7*, or *TMEM165*, alterations in both N-glycosylation and GAG biosynthesis were detected (Foulquier et al., 2012; Edvardson et al., 2013; Ashikov et al., 2018; Dubail et al., 2018). This demonstrates that it could be useful to look for glycosylation defects in patients with CMD to help refine the diagnosis.

## Therapeutic Approaches

As for most of the skeletal and connective tissue disorders, treatments for CMD patients are still restricted to physiotherapy, orthopedic surgery, symptomatic treatment, or monitoring for potential complications to slow down or modify disease progression (Briggs et al., 2015; Marzin and Cormier-Daire, 2020). The development of new innovative therapies requires a strong comprehension of the molecular mechanisms leading to those disorders, mostly through extensive phenotypic analyses of *in vitro* and/or *in vivo* models. This is well exemplified by the use of N-acetylcysteine as a new therapeutic approach for patients with DTD. DTD is caused by pathogenic variants in a gene coding for SLC26A, a cell membrane sulfate-chloride antiporter, resulting in defective sulfate uptake leading to low cytosolic sulfate and subsequently PG undersulfation (Rossi et al., 1998). N-Ac, acting as an intracellular sulfate source for macromolecule sulfation, was thus tested, and preclinical studies in DTD mouse model showed promising results (Monti et al., 2015). N-Ac is currently being tested in DTD. Another example is given by TMEM165. Although the function of TMEM165 is still not completely understood, many observations suggest that TMEM165 has a role in Mn2+ homeostasis in the Golgi. As Mn2+ is a cofactor of glycosyltransferases, impaired Golgi Mn2+ homeostasis in the TMEM165-deficient patient is most likely responsible for the glycosylation defects. Strengthening this hypothesis, experiments performed *in vitro* have demonstrated that Mn2+ supplementation suppressed the glycosylation defects, and Mn2+ supplementation was proposed as potential new therapy for TMEM165-CDG patients (Dulary et al., 2017).

## Concluding Remarks

The recent evolution of genomic technologies has allowed a big step forward in the comprehension of pathophysiological mechanisms leading to rare genetic disorders, including skeletal disorders and the group formed by CMD. Studies of this latter group of pathology have proved a central role of GAG biosynthesis in the pathogenesis of these rare disorders. GAG biosynthesis has characteristic biochemical properties and affects many processes such as embryonic development and connective tissue formation and functions. It is also a complex and tightly regulated process that is still not yet completely clarified. Studies performed on patient samples, cell cultures, and animal models for human CMD have provided new insights on the GAG synthesis and on the physiologic functions of GAG in cartilage, bone, and connective tissues. However, further comprehensive approaches to the molecular pathogenesis involving GAG chains, in association or not with other glycosylation defects, are required to facilitate the development of new biomarkers for clinical screenings and innovative therapeutics for these diseases.

## Author Contributions

JD and VC-D have contributed to the writing of the manuscript. All authors contributed to the article and approved the submitted version.

## Conflict of Interest

The authors declare that the research was conducted in the absence of any commercial or financial relationships that could be construed as a potential conflict of interest. The reviewer AR declared a past co-authorship with one of the authors VC-D to the handling editor.

## Abbreviations

C4ST: chondroitin-4-O-sulfotransferase
C6ST: chondroitin-6-O-sulfotransferase
CANT1: calcium-activated nucleotidase 1
CDG: congenital disorders of glycosylation
CHSY: chondroitin synthase
CMD: chondrodysplasia with multiple dislocations
CS: chondroitin sulfate
CSGalNAcT: chondroitin N-actelygalactosaminyltranferase
D4ST: dermatan-4-O-sulfotransferase
DD: Desbuquois dysplasia
DES: dermatan sulfate epimerase
DS: dermatan sulfate
DTD: diastrophic dysplasia
DTDST: diastrophic dysplasia sulfate transporter
ECM: extracellular matrix
EDS: Ehlers-Danlos syndrome
ER: endoplasmic reticulum
EXT: exostosin
GAG: glycosaminoglycan
Gal: galactose
GalNAcT-I, β1: 4-N-acetylgalactosaminyltransferase-I
GalT-II, β1: 3-galactosyltransferase-II
GalT-I, β1: 4-galactosyltransferase-I
GalNAc: N-acetylgalactosamine
GlcNAc: N-acetylglucosamine
GlcUA: glucuronic acid
gPAPP: Golgi resident phosphoadenosine phosphate phosphatase
IdoUA: iduronic acid
IMPAD1: inositol monophosphate domain-containing protein 1
KS: keratan sulfate
PAP: phosphoadenosine phosphate
PAPS: 3′-phosphoadenosine 5′-phosphosulfate
PG: proteoglycan
UDP: uridine diphosphate
Xyl: xylose
XYLT: xylosyltransferase.
